# Characterization of Diversity and Probiotic Efficiency of the Autochthonous Lactic Acid Bacteria in the Fermentation of Selected Raw Fruit and Vegetable Juices

**DOI:** 10.3389/fmicb.2018.02539

**Published:** 2018-10-23

**Authors:** Xinxing Xu, Dongsheng Luo, Yejun Bao, Xiaojun Liao, Jihong Wu

**Affiliations:** ^1^Beijing Advanced Innovation Center for Food Nutrition and Human Health, College of Food Science and Nutritional Engineering, China Agricultural University, Beijing, China; ^2^National Engineering Research Center for Fruit and Vegetable Processing, Beijing, China; ^3^Key Laboratory of Fruit and Vegetable Processing, Ministry of Agriculture, Beijing, China; ^4^Beijing Key Laboratory for Food Non-thermal Processing, Beijing, China

**Keywords:** autochthonous lactic acid bacteria, microbial diversity, fermentation, fruit and vegetable juice, probiotic viability

## Abstract

The diversity of indigenous lactic acid bacteria (LAB) in fermented broccoli, cherry, ginger, white radish, and white-fleshed pitaya juices was analyzed using culture-independent and -dependent approaches. The major properties of selected probiotic strains, including dynamic variations in pH, viable cell counts, antibiotic resistance, bacterial adhesion to hydrophobic compounds, and survivability during simulated gastrointestinal transit, were investigated using broccoli as the fermentation substrate. In broccoli and ginger juices, the genus *Lactobacillus* occupied the dominant position (abundances of 79.0 and 30.3%, respectively); in cherry and radish juices, *Weissella* occupied the dominant position (abundances of 78.3 and 83.2%, respectively); and in pitaya juice, *Streptococcus* and *Lactococcus* occupied the dominant positions (52.2 and 37.0%, respectively). *Leuconostoc mesenteroides, Weissella cibaria/soli/confusa, Enterococcus gallinarum/durans/hirae, Pediococcus pentosaceus, Bacillus coagulans*, and *Lactococcus garvieae/lactis* subspecies were identified by partial 16S rRNA gene sequencing. In general, the selected autochthonous LAB isolates displayed no significant differences in comparison with commercial strains with regard to growth rates or acidification in fermented broccoli juice. Among all the isolates, *L. mesenteroides* B4-25 exhibited the highest antibiotic resistance profile (equal to that of *L. plantarum* CICC20265), and suitable adhesion properties (adhesion of 13.4 ± 5.2% ∼ 36.4 ± 3.2% and 21.6 ± 1.4% ∼ 69.6 ± 2.3% to ethyl acetate and xylene, respectively). Furthermore, *P. pentosaceus* Ca-4 and *L. mesenteroides* B-25 featured the highest survival rates (22.4 ± 2.6 and 21.2 ± 1.4%, respectively), after simulated gastrointestinal transit. These results indicated a high level of diversity among the autochthonous bacterial community in fermented fruit and vegetable juices, and demonstrated the potential of these candidate probiotics for applications in fermentation.

## Introduction

Fermented fruit and vegetable juices (FVJs) containing lactic acid bacteria (LAB) are important research targets with regard to providing additional value and choices for vegetarians and individuals with lactose intolerance (Di Cagno et al., 2013). Abundant sources of autochthonous LAB exist in the spontaneous fermentation of fruits and vegetables, which is carried out in exclusive uncontrollable environmental conditions (Sanni, 1993; Steinkraus, 1997). Researchers have explored the use of indigenous LAB strains isolated from food materials and their addition to more complex food systems, with the aim of improving the quality characteristics and functional properties of the end products (Galvez et al., 2007; Di Cagno et al., 2009a; Ong et al., 2012). For instance, autochthonous LAB obtained by the fermentation of mango juice can be employed to compensate for the loss of antioxidant substances, increase the contents of nutrients such as organic acids and mannitol, and provide better sensory characteristics such as acidity and sweetness (Liao et al., 2016). However, different probiotics have different survival characteristics and functional performance in various juices. A previous screening study was conducted to determine which, among apple, grape, and orange juices, was the best substrate for the growth of *Lactobacillus* strains with respect to bacterial viability, superoxide dismutase activity, folate production, and hedonic characteristics (Espirito-Santo et al., 2015). Researchers have revealed that indigenous probiotics isolated from raw materials have an inherent stability, which may contribute to improving the survival rate and persistence observed in food matrices (Ong et al., 2012; Reina et al., 2015). Therefore, the characterization and identification of indigenous probiotics from various FVJs could provide diverse microbiological resources with enhanced fermentative capabilities for the manufacturing of products with greater stability and production efficiency (Gibbons and Rinker, 2015; Bokulich et al., 2016).

The combined utilization of culture-independent and -dependent analyses is useful for profiling complex microbial taxonomic communities and assessing the viability of cultivable microbial populations (Kesmen et al., 2012; Davis, 2014). High-throughput sequencing has emerged as an innovative culture-independent technique to quantitatively investigate the biodiversity of microbial communities in foods, and has been proven to be reliable in the study of dominant, as well as minor, microbial populations (Medina et al., 2016). The method that is based on the cultivation of microorganisms in selective media has a specific advantage in that it can yield single colonies of the bacteria that are present in the fermentation ecosystem, enabling their selective isolation, cultivation, and identification (Ellis et al., 2003). However, the majority of previous studies that used these two methods have focused on fermented seafoods, soybean paste, kimchi, wine, sourdough, soil etc. (Nam et al., 2012; Park et al., 2012; Adewumi et al., 2013; Ercolini et al., 2013; Jung et al., 2013; Pinto et al., 2015), whereas few studies have assessed fermented FVJs. The studies that assessed fermented FVJs employed a single method and/or used commercial LAB cultures (Aneja et al., 2014; Nicomrat and Chamutpong, 2016). Moreover, the environments inside different fermented FVJs with surface microorganisms are markedly dissimilar with regard to multiple factors, including the variety of carbohydrates/carbon sources and nutritional compositions, and discrepancies in the initial pH. In addition, the presence of amino acids, vitamins, dietary fibers, phenolic compounds, mixed oligosaccharides, and other bioactive substances gives rise to food substrates that have probiotic properties, such as antioxidant activity, antiproliferative effects on cancer cells, and the capacity to stimulate the growth of *Lactobacillus* and *Bifidobacterium* species (Granato et al., 2010; Nematollahi et al., 2016). Therefore, by selecting a wide diversity of raw materials, we were able to investigate dissimilarities in their microbial profiles and identify promising LAB strains in these FVJs. In this study, cherry (Jacob et al., 2003; Chaovanalikit and Wrolstad, 2004; Kim et al., 2005), white-fleshed pitaya (Wichienchot et al., 2010; Garcia-Cruz et al., 2017), white radish (Hashimoto et al., 2006; Lee et al., 2012; Kaymak et al., 2015), broccoli (Keck et al., 2003; Moreno et al., 2006; Berenbaum, 2014; Armah et al., 2015), and ginger (Kruth et al., 2004; Palatty et al., 2013; Daily et al., 2015), which have been shown to exhibit a great variety of bioactive characteristics, were chosen as substrates for the isolation of LAB strains and follow-up testing.

To the best of our knowledge, detailed investigations of variations in the indigenous bacterial community in fermented FVJs are limited. The objective of this study was to characterize and identify the microbial diversity of fermented non-pasteurized fresh FVJs using culture-independent and -dependent methods and to determine whether isolated indigenous microbes habituated on the surface of fruits and vegetables could be successfully cultivated and used for inoculating commercial products. In addition, we aimed to identify ideal substrates that could be selected for delivering such isolated LAB.

## Materials and Methods

### Sampling

Broccoli (*Brassica oleracea*), cherry (*Prunus avium*), ginger (*Zingiber officinale*), white radish (*Raphanus sativus*), and white-fleshed pitaya (*Hylocereus undatus*) were collected from a local market (Beijing, China) and stored at 4°C prior to use. The fermented samples were prepared according to a previously reported method (Di Cagno et al., 2016). Fifty grams of each sample were suspended in 50 mL MRS broth separately and fermented for 48 h at 37°C in an anaerobic incubator (LAI-3-T, Shanghai Longyue Instruments Equipment Co., Ltd., Shanghai, China). Unfermented samples were prepared according to a previously described method (Di Cagno et al., 2009a). Ten grams of each sample were suspended in 90 mL of sterile sodium chloride (0.9% w/v) solution and homogenized (FB-110Q, Shanghai Litu Mechanical Equipment Engineering Co., Ltd., Shanghai, China) for 2 min at room temperature.

### Microbial Diversity Analysis

The fermented juices were successively filtered through 0.45- and 0.22-μm membranes. Microbial DNA was extracted using the E.Z.N.A.^®^ Soil DNA Kit (Omega Bio-tek, Norcross, GA, United States) according to the manufacturer’s protocols. The final concentration and purification of DNA were determined using a NanoDrop 2000 UV-vis spectrophotometer (Thermo Scientific, Wilmington, DE, United States), and DNA quality was checked via 1% agarose gel electrophoresis. The V3 and V4 hypervariable regions of the bacteria 16S rRNA genes were amplified with primers 338F (5′- ACTCCTACGGGAGGCAGCAG-3′) and 806R (5′-GGACTACHVGGGTWTCTA AT-3′) using a thermocycler polymerase chain reaction (PCR) system (GeneAmp 9700; ABI, Carlsbad, CA, United States). PCR was conducted using the following program: denaturation for 3 min at 95°C; 27 cycles of 30 s at 95°C, annealing for 30 s at 55°C, and elongation for 45 s at 72°C; and a final extension at 72°C for 10 min. The PCR was performed in triplicate, with 20-μL reactions containing 4 μL of 5 × FastPfu Buffer, 2 μL of 2.5 mM 2′-deoxynucleoside 5′-triphosphate (dNTPs), 0.8 μL of each primer (5 μM), 0.4 μL of FastPfu Polymerase, and 10 ng of template DNA. The PCR products were extracted from a 2% agarose gel and further purified using an AxyPrep DNA Gel Extraction Kit (Axygen Biosciences, Union City, CA, United States) and quantified using a QuantiFluor^TM^-ST fluorometer (Promega, Madison, WI, United States) according to the manufacturer’s protocol. Purified amplicons were pooled in equimolar ratios and paired-end sequenced (2 × 300) on an Illumina MiSeq platform (Illumina, San Diego, CA, United States) according to the standard protocols of Majorbio Bio-Pharm Technology Co., Ltd. (Shanghai, China).

The raw fastq files were demultiplexed, quality-filtered using the Trimmomatic tool, and merged using FLASH software with the following criteria: (i) the reads were truncated at any site that received an average quality score of less than 20 over a sliding window of 50 bp. (ii) Primers were exactly matched allowing two-nucleotide mismatching, and reads containing ambiguous bases were removed. (iii) Sequences with overlap longer than 10 bp were merged according to their overlap sequence. Operational taxonomic units (OTUs) were clustered with 97% similarity cutoff using UPARSE software (version 7.1^1^), and chimeric sequences were identified and removed using UCHIME. The taxonomy of each 16S rRNA gene sequence was analyzed using RDP classifier algorithm^2^ by reference to the Silva (SSU123) 16S rRNA database with a confidence threshold of 70%.

### Isolation and Identification of LAB Strains

The isolation of strains was carried out according to a previously described method (Di Cagno et al., 2009a). Each sample was serially diluted 10^-1^∼ 10^-7^-fold with sterilized saline. Thereafter, 100-μL dilutions were spread onto MRS agar plates. After incubation at 37°C for 48 h under anaerobic conditions, colonies with different morphotypes from the highest dilutions were collected in MRS broth supplemented with 20% glycerol, and stored at -80°C for further analyses (Park et al., 2016). Gram-positive, catalase-negative, non-motile rods and cocci were cultivated in MRS broth at 37°C for 24 h, and then re-streaked onto MRS agar. Identification of the screened LAB strains was performed by sequencing the 16S rDNA gene. Genomic DNA of selected LAB strains was extracted from cultures grown at 37°C for 24 h in MRS broth using TRIzol reagent (Tiangen Biotechnology Co., Ltd., Beijing, China) and amplified by PCR using two universal primers, namely, 27F (5′-AGA GTT TGA TCC TGG CTC AG-3′) and 1492R (5′-GGY TAC CTT GTT ACG ACT T-3′) (Ding et al., 2017). Fifty microliters of each PCR mixture contained: 4 μL 2.5 mM of dNTPs, 1 μL of both forward and reverse primer, 2 μL template, and 0.5 μL 5 U of Taq DNA polymerase [Takara Biomedical Technology (Beijing) Co., Ltd., Beijing, China], in 5 μL supplied buffer. The expected amplicons of about 1465 bp after amplification with the primer pair were eluted from the gel and purified. PCR products were sequenced by Majorbio Biotechnology Co., Ltd. (Shanghai, China), and the sequences were compared with the sequence database in the National Center of Biotechnology Information (NCBI) using the basic local alignment search tool (BLAST^3^) to identify the strains at the species level (Altschul et al., 1990). The sequences of highly homologous type strains were downloaded from GenBank database, and a phylogenetic study was carried out with MEGA version 5 (Tamura et al., 2011). The obtained sequences were lined up by ClustalX software (Kohli and Bachhawat, 2003), and the neighbor-joining algorithm was used to construct a phylogenetic tree based on distance estimates calculated by the Kimura-2 parameter, which includes a bootstrap test with 1000 replicates (Saitou and Nei, 1987).

### Determination of pH and Viable Cell Count in Fermented Broccoli Juice

Broccoli was blended with purified water (1:3, g/mL) and then pasteurized for 5 min at 80°C. From the MRS broth cultures of the selected LAB strains (10^9^ CFU/mL), 0.4 mL was centrifuged for 10 min at 10000 × *g*, and the resulting precipitate of bacteria was added to 40 mL broccoli juice to obtain an initial count of mesophilic LAB of 10^8^ CFU/mL in the final juice sample. Fermentation experiments were conducted in 50-mL sterile centrifuge tubes, each containing 40 mL juice. The juice was then incubated for 48 h at 37°C in an anaerobic incubator (LAI-3-T, Shanghai Longyue Instruments Equipment Co., Ltd., Shanghai, China). Meanwhile, the progress of fermentation was monitored every 12 h by quantifying the colony forming units (CFUs) on MRS agar plates using the standard method of decimal dilution and measuring the pH using a pH meter (Medidor pH basic 20, Crison Instruments, Spain) (Di Cagno et al., 2008).

### Antibiotic Resistance

The standard disk diffusion assay was used to determine the sensitivity or resistance of LAB to conventional antibiotics. Paper disks containing ampicillin (10 μg), penicillin (10 μg), amoxycillin (10 μg), norfloxacin (10 μg), levofloxacin (5 μg), gentamicin (120 μg), streptomycin (10 μg), amikacin (30 μg), and erythromycin (15 μg), which were purchased from Solarbio Technology Co., Ltd. (Beijing, China), were employed for the antibiotic resistance tests (Lee et al., 2014). From the MRS broth culture of each one of the test strains, 100 μL was mixed with 8 mL of liquid MRS agar, over-layered on a pre-solidified agar plate and allowed to solidify, and then disks were aseptically placed onto the center of plates using sterile forceps. The plates were incubated for 48 h at 30°C in an anaerobic chamber. The results were recorded according to the interpretive category defined by the Clinical and Laboratory Standards Institute (CLSI) (Sharma et al., 2017). The tests were carried out in triplicate.

### Determination of Hydrophobicity

The hydrophobicity of LAB isolates was assessed using a modified version of a previously reported method (Bautista-Gallego et al., 2013). Ethyl acetate and xylene were used as the hydrophobic substances for the hydrophobicity assays according to the recommendations of previous reports (Vanhaecke and Pijck, 1988; Guo et al., 2010). One milliliter of fermented broccoli juice (10^9^ CFU/mL) was centrifuged for 15 min at 8000 × *g* and washed twice with phosphate-buffered saline (PBS). The pellet was resuspended in PBS, and the optical density was assessed at 600 nm (*A*_0_). Equal proportions of ethyl acetate and xylene were blended with the bacterial cells and vortexed for about 5 min, incubated for 1 h at room temperature, and the optical density was assessed at 600 nm (*A*_1_). The capability of the bacteria to adhere to the hydrophobic compound (BATH) was calculated as follows:

$$BATH\%=[(A_{0}-A_{1})/A_{0}]\times 100$$

### Assessment of the Survivability of LAB in Simulated Gastrointestinal Transit

The survivability of the LAB isolates in the presence of artificial gastrointestinal juices was measured by the method described by Baruah et al. (2017): (i) First, 1 mL fermented broccoli juice (10^9^ CFU/mL) was centrifuged for 10 min at 8000 × *g* and the pellet was washed twice with sterile PBS before being resuspending in 10 mL of simulated gastric juice (GJ), and incubated for 90 min at 37°C. The GJ consisted of pepsin (1000 U/mL) in PBS, with the pH adjusted to 2.5 with 10% hydrochloric acid. (ii) The solution was then centrifuged for 10 min at 8000 × *g*, the supernatant was removed, and the precipitate was re-suspended in 10 mL simulated duodenal juice (DJ) and incubated for 10 min at 37°C. The DJ was composed of 1% (w/v) bile salts, and the pH was adjusted to 8.0 with 1M NaOH. (iii) The solution was then centrifuged for 10 min at 8000 × *g*, the supernatant was removed, and the precipitate was re-suspended in 10 mL simulated intestinal juice (IJ) and incubated for 120 min at 37°C. The intestinal fluid was composed of 0.3% (w/v) bile salts and 1000 U/mL of trypsin solution, and its pH was adjusted to 8.0. The viable bacterial cell counts were determined by serial dilution in physiological saline solution at the beginning of each step and at the end of the last step.

### Data Analysis

All experiments were performed in triplicate. Data fitting was performed using the software Statistica for Windows ver. 10. Data were also analyzed via one-way ANOVA and Tukey’s test (*P* < 0.05). The results are expressed as the mean ± standard deviation. Each of the bars represents the standard deviation from the mean.

## Results and Discussion

### The Structure of the Uncultured Microbial Community in Fermented Samples

**FIGURE 1:**
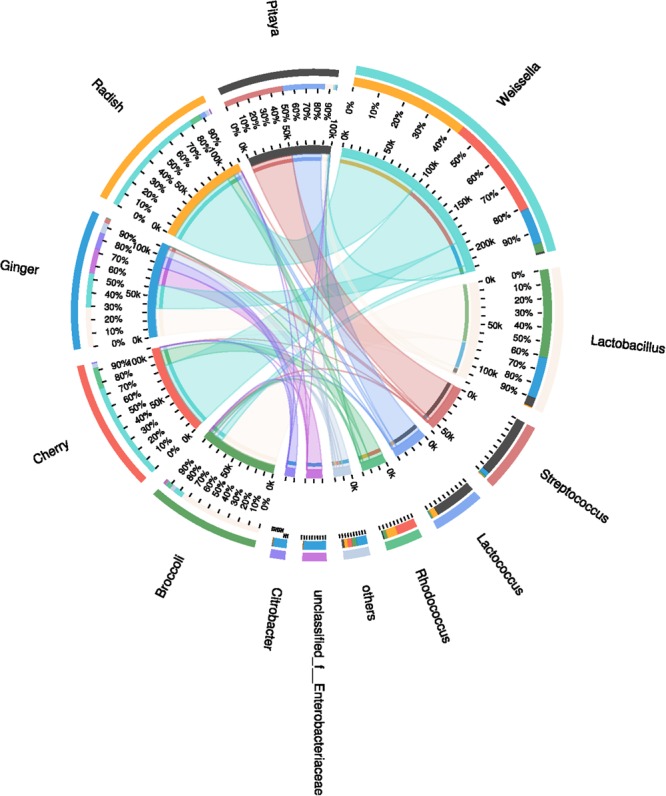
Distribution of the microbial community for each sample at the genus level. The data were visualized by Circos. The small semi-circle **(left)** shows the composition and abundance of species in each sample. The large semi-circle **(right)** shows the distribution ratio of samples within the dominant species, and the width of the bars for each genus indicates the relative abundance of that genus in the sample. From the outer circle to the inner circle, the outer circle shows the different samples and species using specific colors, and the length is related to the distribution proportion. The second circle shows the percentage. In the innermost circle, the two ends of each chromatic stripe connect the sample with the dominant genus; the width of the stripe at the endpoint represents the abundance and distribution proportion, and the numerical value outside the circle represents the abundance values of the corresponding samples and species.

Throughout the five different samples, a total of 246 OTUs at a distance of 3% was obtained, with an average of 98 OTUs in each sample, including repetitive OTUs. Rarefaction analysis demonstrated the abundances in the different samples, and rarefaction curves for a similarity of 97% indicated that the sufficient coverage of sequencing could account for the majority of the bacterial diversity within each sample. The coverage indices, which were greater than 99%, also indicated that the microbial community was reflected accurately (Wang et al., 2017). A total of 16 phyla were detected via taxonomic analyses. The five most abundant phyla were *Bacteroidetes* (0.2%), *Cyanobacteria* (0.4%), *Actinobacteria* (6.2%), *Proteobacteria* (8.6%), and *Firmicutes* (84.6%). A total of 154 bacterial genera were identified. The most abundant genera were characterized to elucidate which might be the most important bacteria present in the fermented FVJs ecosystem. The relative abundances (%, abundances >5%) and distributions of the dominant microorganisms in the different juices, as determined via the genus analysis, can be clearly ascertained in Figure 1. The microbiota was found to be almost exclusively dominated by members of the phylum *Firmicutes*; in particular, of the five principal OTUs in all five materials, three belonged to LAB, namely *Weissella* (46.0%), *Lactobacillus* (24.2%), and *Streptococcus* (12.1%), and the other two corresponded to *Rhodococcus* (6.7%) and *Enterobacteriaceae* (4.6%). However, the dominant genera were different in each juice. In broccoli juice, *Lactobacillus* occupied the dominant position with an abundance of 79.0%, and the number of 125 OTUs was the highest recorded among the five raw materials, which may be correlated to the specific structure of the broccoli flowering head. *Lactobacillus* and *Weissella* were the most abundant genera in ginger juice, with abundances of 30.3 and 25.8%, respectively. In cherry and radish juices, *Weissella* occupied the dominant position (78.3 and 83.2%, respectively), whereas *Streptococcus* and *Lactococcus* occupied the dominant position (52.2 and 37.0%, respectively) in pitaya juice. Naturally occurring microbial populations in food ecosystems are responsible for spontaneous fermentation that leads to a variety of traditionally fermented products, which represent a valuable reservoir of novel strains of environmental origin (Tamang et al., 2016). In this study, high-throughput sequencing enabled the analysis of the microbial community as a whole, whereas culture techniques provide isolates for further applications (Perez-Cataluna et al., 2018). We observed that the dominant genera in the different fermented juices varied, which was partially congruent with the results of dominant genera previously identified within the microbiota of banana, kimchi, cucumber, tomato, chard, and other fruits and vegetables (Choi et al., 2003; Lee et al., 2004; Di Cagno et al., 2009a; Nicomrat and Chamutpong, 2016). Such differences in diversity were probably associated with the geographical location, harvesting season, storage position, processing techniques used, and other complex and various factors (Yoon et al., 2017). For example, the distribution of genera of *Enterobacteriaceae* differed significantly between the samples, and their relative abundance in ginger juice reached 19.0% whereas it was less than 0.5% in the other four samples. Some species in the *Enterobacteriaceae* family are known to be pathogenic or opportunistic. The ubiquity of *Enterobacteriaceae* genera in the studied ginger samples may be ascribed to unhygienic handling, inappropriate processing or storage conditions in the market (Stoops et al., 2016). Evidence suggests that the structural diversity of bacterial communities is closely associated with the organoleptic attributes, nutrients and, the quality of the fermented products (Liu and Tong, 2017). For instance, some species in the genus *Weissella* have potential as probiotics, owing to their ability to produce exopolysaccharides (e.g., *Weissella cibaria* and *Weissella confusa*) (Fusco et al., 2015). Moreover, a number of studies have reported the dominant effective microbes present in traditionally fermented pickles, fermented dough, yogurt, and fermented wine, as well as the production of foods fermented with LAB strains from their natural microbiota (Ben Omar and Ampe, 2000; Pinto et al., 2015; Fan et al., 2017; Motato et al., 2017). These analyses, highlighting the diversity and richness of microbial communities among the fermented FVJs, provided the foundation for the separation of LAB isolates (Pinto et al., 2015).

### Variance Analysis of Samples and Dominant Species

**FIGURE 2:**
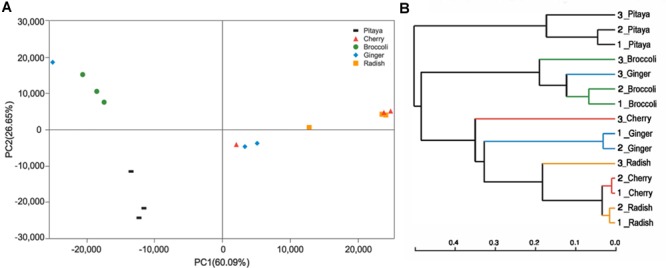
Variance analysis of the bacterial communities among the fermented juices. **(A)** PCA plots indicate the abundance of diverse bacteria in the fermented juices. The first principal component (PC1) and second principal component (PC2) shows 26.65 and 60.09%, respectively, of the variance in the unweighted Unifrac metrics. Each point represents the microbiota from a single sample. **(B)** Hierarchical clustering of the group means based on UPGMA.

**FIGURE 3:**
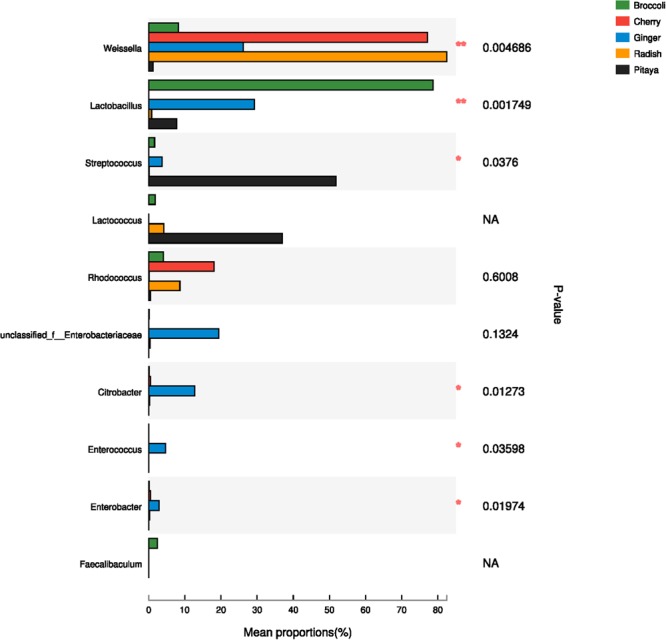
Comparison of dominant genera in five samples using one-way ANOVA. In the vertical axis, the different identified bacteria genera are depicted; the length of the corresponding column indicates the average of the relative abundance of the genus in the different samples, ^∗∗^*P* < 0.01, ^∗^*P* < 0.05; NA, not available.

The calculated values of the Shannon index of microbial diversity for the fermented juices (α-diversity) showed no significant differences, as demonstrated by an independent *t*-test. Moreover, the abundance matrix that was obtained from the fermented juices was subjected to principal component analysis (PCA) and hierarchical clustering analysis (β-diversity) (Figures 2A,B). The differences in the distributions among the fermented samples did not indicate significant dissimilarities, and the individual variations that were observed may be related to the preparation processes. Clustering of the various samples, which was based on the unweighted pair-group method with arithmetic mean (UPGMA), also did not show a statistically significant difference in the microbial diversity between pitaya, broccoli, ginger, cherry, and radish. Previous knowledge regarding such microbial biodiversity was mainly based on studies that assessed the processing of products such as olives, fermented sausage, and fermented cabbage (Giello et al., 2018; Medina et al., 2018; Wang and Shao, 2018). Comparatively, there is little research on the microbial composition of fermented FVJs using high throughput sequencing. In our study the microbial community structure in different samples showed no significant differences in α- and β- diversity; however, the dominant genera were variable. This contradicts with the results of the microbial profiles reported in other fermented vegetables, which significantly differed based on their region of origin and raw materials used (Peng et al., 2018). The results of difference analysis concerning the abundance of predominant genera in the five samples are depicted in Figure 3. The significance testing used strict statistical methods to detect obvious differences between genera on the basis of the data on abundances in the communities. The abundance of *Weissella* and *Lactobacillus* presented highly significant differences among the five samples (*P* < 0.01). In addition, *Streptococcus, Citrobacter, Enterococcus*, and *Enterobacter* also displayed significant differences between the samples (*P* < 0.05). *Weissella* has occasionally been found in fermented foods in comparison with *Lactobacillus* (Karovicova and Kohajdova, 2005); these typical genera used in fermentation may promote various quality properties and are valuable sources of functional ingredients. For instance, *W. cibaria* RBA12 from pomelo can generate dextran and the survival of *Staphylococcus aureus* can be suppressed by *Lactobacillus rhamnosus* LOCK900 from carrot juice (Trząskowska and Gasentzer, 2016; Baruah et al., 2017). The verification of microbial diversity of food-inherent ecosystems is essential for revealing the natural processes and reconstructing such ecosystems under optimized and controlled conditions.

### Identification and Typing of Isolated LAB Strains

**FIGURE 4:**
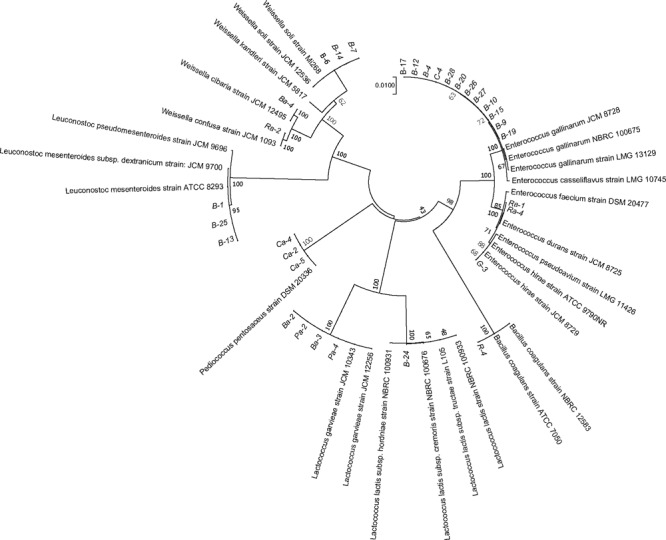
Phylogenetic tree constructed with sequences of the partial 16S rRNA gene of selected LAB strains. The 16S sequences of the isolated bacteria are aligned with reference strains. The data of type strains of related species were from GenBank database. Branch lengths are proportional to distance. Bootstrap values are indicated near the internodes. B, R, C, G, and P represent juices of broccoli, radish, cherry, ginger, and pitaya, respectively; Ba, Ra, Ca, Ga, and Pa represent juices of broccoli, radish, cherry, ginger, and pitaya after fermentation, respectively.

Lactic acid bacteria strains were isolated from fermented FVJs and freshly squeezed juice without sterilization. The number of strains in each sample was determined according to macroscopic (colony morphology) and microscopic (cell morphology) characteristics. After confirmation of negative catalase reaction, Gram staining, and 16S rRNA sequencing analysis, the presumptive mesophilic LAB present in the highest dilution of the different fermented juices were identified. Phylogenetic relationships of the isolates together with representative 16S bacterial sequences were analyzed using the neighbor-joining method (Saitou and Nei, 1987). The resulting tree showed that the 32 isolates could be classified into six clusters on the basis of similarities in 16S rRNA sequences (Figure 4), namely *Leuconostoc* (3 isolates), *Weissella* (5 isolates), *Lactococcus* (5 isolates), *Pediococcus* (3 isolates), *Enterococcus* (15 isolates), and *Bacillus* (1 isolates). All isolates of different genera were separated into unique clusters. Notably, the similarity could be visualized among phylogenetically related isolates in Figure 4. At the similarity level of 70%, the lowest percentage of the isolates was grouped in genus *Enterococcus*, three isolates of *Leuconostoc mesenteroides* were closely related to *L. mesenteroides* ATCC 8293 with 95% identity, and other isolates were put in separate branches of the tree and showed 100% of identity with related type strain 16S rRNA sequences. The following species were identified for each sample: broccoli, *L. mesenteroides* (3 isolates), *Weissella cibaria/soli* (4 isolates), *Enterococcus gallinarum* (11 isolates), *Lactococcus garvieae/lactis subspecies* (3 isolates); cherry, *Pediococcus pentosaceus* (3 isolates), *E. gallinarum* (1 isolates); radish, *W. confusa* (1 isolate), *Enterococcus durans* (2 isolates), *Bacillus coagulans* (1 isolate); pitaya, *Lactococcus garvieae* (2 isolates); ginger, *Enterococcus hirae* (1 isolate). *Lactococcus lactis* subspecies isolate B-24 from broccoli juice needed a further identification based on the 16S rRNA and *recA, groEL* genes (Le Bourgeois et al., 2015). The results of the culture-dependent analysis demonstrated that species in fermented radish and pitaya juices with the highest concentration were in accordance with the most highly abundant species detected by culture-independent analysis, namely, *Weissella* and *Lactococcus* species, respectively. However, the results of *P. pentosaceus* in fermented cherry juice (approximately 10^6^ CFU/mL) as well as *Lactococcus* and *Weissella* (approximately 10^7^ CFU/mL) in fermented broccoli juice did not match the high throughput sequencing results. The dominant microbiota in fermented broccoli and ginger juices were *Lactobacillus* species, but no isolates from this genus were detected in the highest dilutions of fermented ginger juice. Probably the necessary conditions for successful isolation of the different *Lactobacillus* species might not be fully efficient with MRS as the selecting medium, since some species of this genus require enrichment conditions for their successful isolation from environmental samples. This inconsistency has also been observed during the detection of potential foodborne pathogens during the kimchi elaboration process (Lee et al., 2017). This phenomenon may be ascribed to the facts that not all the isolates in the different dilutions were identified and culture-independent analyses did not discriminate between live and dead microbial cells (Fusco and Quero, 2014; Liu and Tong, 2017). In addition, changes in the fermentation conditions also played a role in the distribution of colonies. For example, it has been shown that the *Pediococcus* species can be detected in table olives through culture-independent analysis, but cannot be isolated which could be due to their low survival rate in acidic conditions (Sanchez et al., 1995). Species in the genus *Enterococcus* can play a positive role in various fermented products and have attracted more attention in recent years, than the normally relatively common species in LAB groups (M’hir et al., 2012). Although species mainly from humans and domestic animals have been studied in some detail, limited information is available on plant-associated species. Figure 4 shows that *E. gallinarum, E. durans*, and *E. hirae* were identified in the fermented juices. Even though *Enterococcus* species are considered indicators of fecal contamination (e.g., in water), or even as potentially pathogenic microorganisms, they possess many desirable properties, such as improvements in sensory characteristics, natural preservation, and health-related benefits, that could increase the value of vegetable-based fermented foods (Ben Omar et al., 2004). The heterogeneous nature of fermented products, with variations in microbial diversity, quality, and properties, requires the exploitation of appropriate starter cultures to initiate fermentation and obtain consistent products with acceptable quality. Undoubtedly, the identification of relevant strains can provide the foundation of a mixed fermentation starter for the elaboration of compound juices.

### Dynamic Variance in Viable Cell Counts and pH in Fermented Broccoli Juice

**FIGURE 5:**
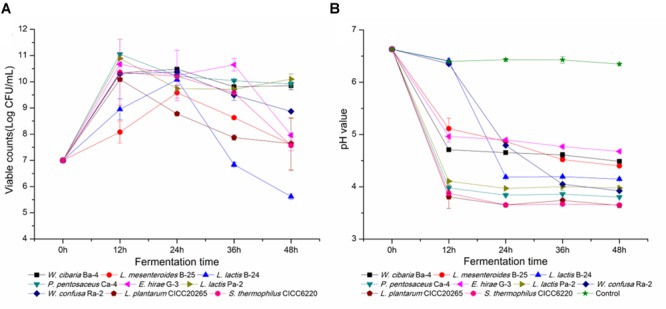
Viable cell counts and pH values throughout the fermentation of broccoli juices incubated with the nine selected LAB strains. **(A,B)** Represent the change in viable cell counts and pH, respectively.

*Weissella cibaria* Ba-4, *L. mesenteroides* B-25, *L. lactis* subspecies B-24, *P. pentosaceus* Ca-4, *E. hirae* G-3, *L. garvieae* Pa-2, and *W. confusa* Ra-2 were selected for further investigation. Each of these strains belonged to different clusters among the various LAB strains that were identified. To assess their adaptation to broccoli juice, dynamic variations in pH and viable cell counts were determined and compared with those of the commercial starters, *L. plantarum* CICC20265 and *S. thermophilus* CICC6220. The cell densities of all the autochthonous strains increased from 7.0 Log CFU/mL to values that ranged from 10.2 ± 0.39 to 11.0 ± 0.58 Log CFU/mL. Overall, the stationary growth phase was reached after 18 h fermentation at 37°C, when both the commercial and the autochthonous strains reached a cell density of 10.52 ± 0.37 Log CFU/mL (Figure 5A). Based on previous research, we know that allochthonous strains tend to demonstrate poor growth characteristics in comparison with autochthonous isolates (Di Cagno et al., 2009a), as has been reported for fermented carrots (8.57/7.62 Log CFU/mL), French beans (8.95/8.08 Log CFU/mL), marrows (8.48/7.40 Log CFU/mL), mangoes (10.33/7.71 Log CFU/mL), and tomatoes (9.8/8.52 Log CFU/mL) (Di Cagno et al., 2008, 2009a,b; Liao et al., 2016). Although almost a similar behavior was observed for the commercial species, the indigenous isolates may certainly have influenced the fermentation and the characteristics of the final product.

Furthermore, dynamic changes in pH were directly associated with the cell density of LAB. As shown in Figure 5B, the pH of *L. lactis* B-24 and *W. confusa* Ra-2 cultures decreased significantly after 18 h of fermentation in comparison with broccoli juice fermented without a starter (pH 6.63 ± 0.02), and the average pH of *L. plantarum* and *S. thermophilus* fermented juices was as low as 3.65 ± 0.12. *P. pentosaceus* Ca-4 (pH 3.83 ± 0.24) and *L. garvieae* Pa-2 (pH 3.98 ± 0.06) reflected the best acidification characteristics in fermentation. As previously reported, indigenous strains of *L. mesenteroides* can reduce the pH of fermented prickly pear from 6.01 to 4.07, *W. confusa* reduced the pH of fermented peppers from 5.0 to 3.7, and *L. plantarum* reduced the pH of fermented tomato juices from 4.3 to 3.78 (Di Cagno et al., 2009a,b, 2016). However, the strains isolated from broccoli juice did not show any obvious superiority in growth rate and capability to decrease pH, and this probably might be attributed to inherent characteristics of the raw material used for growing the LAB strains (Santo et al., 2011). Previous reports have indicated that *Lactobacillus* and *Bifidobacterium* strains sustain higher viability in orange and pineapple juices in comparison with cranberry juice (Sheehan et al., 2007), and similar results have also been observed for pomegranate juice when different starters were used (Mousavi et al., 2011). Our observations indicated that broccoli juice was appropriate for LAB fermentation, as it enabled a rapid bacterial growth and a sufficient population of viable cells, consistent with the results of tomato, carrot, cabbage, artichokes, and reed beet juices in regards to suitability as a fermentation substrate (Valerio et al., 2006; Rivera-Espinoza and Gallardo-Navarro, 2010; Di Cagno et al., 2013). It would be beneficial to optimize a combination of species isolated from fermented raw fruits and vegetables, with the aim of comprehensive utilization in a wide range of fermented foods.

### Antibiotic Resistance

Lactic acid bacteria strains have been widely used in commercial applications and have been specifically selected to discourage the spread of antibiotic resistance and prevent the exchange of transferable resistance genes (Ouwehand et al., 2016). According to the breakpoints recommended by the European Food Safety Authority (European Food Safety Authority [EFSA], 2012) and the interpretive category defined by CLSI, the antibiotic resistance was shown in Table 1, our results demonstrated that the nine selected isolated strains were all susceptible to amoxicillin (10 μg) and resistant to amikacin (30 μg). Charteris et al. (1998) tested 46 *Lactobacillus* strains from human and dairy sources for susceptibility to 44 antibiotics, and all strains were resistant to 14 antibiotics, including amikacin (30 μg), gentamicin (10 μg), streptomycin (10 μg), and norfloxacin (10 μg) (Charteris et al., 1998), antibiotics that were also assessed in our study. In contrast to this previous report, the nine strains tested in the present study exhibited sensitivity or intermediate susceptibility to gentamicin (10 μg). There have been reports that corroborate our findings regarding the susceptibility of LAB to gentamicin, for example, Jiang et al. (2016) reported the intermediate susceptibility to gentamicin of *Lactobacillus* strains isolated from human milk. Furthermore, isolates belonging to the same species may show several sensitivities to the same antibiotic; for instance, most *Leuconostoc* species tested were resistant to gentamicin (10 μg), but *L. mesenteroides* B-25 was susceptible (Ammor et al., 2007). It has also been demonstrated that the source of indigenous isolates influences the antibiotic resistance; 31 indigenous *Lactobacillus* isolates from curd and human milk showed strong resistance to streptomycin (10 μg) (Sharma et al., 2017). In the three isolates of our study, only an intermediate susceptibility was observed. All the isolates tested in the present study exhibited strong resistance to erythromycin (15 μg) except for *L. lactis* subspecies B-24. However, earlier studies showed that low resistance frequencies (0.7% in each case) among LAB isolates of *Lactobacillus, Pediococcus*, and *Lactococcus* species have potential for probiotic or nutritional use (Klare et al., 2007). In general, previous results have indicated that variations in source, species, inoculum size, incubation temperature and time, and even the test medium can influence the activity of probiotics including the pattern of antibiotic sensitivity (Herrero et al., 1996). The high resistance and sensitivity of LAB strains to a range of antibiotics used in the medical practice is considered highly significant, since there is the probability of transferring antibiotic resistance from LAB strains to other undesirable and detrimental organisms. The ability to transfer antibiotic resistant factors must be considered as an important parameter in the selection of probiotic strains. Most studies on antibiotic resistance that have been conducted so far in LAB have involved members of the genus *Enterococcus*, which occupies a peculiar position among food microorganisms. The *Enterococcus* species play a pivotal role in traditionally fermented foods, but their role as opportunistic pathogen has also been acknowledged. Our results demonstrated that *E. hirae* G-3 showed susceptibility and intermediate susceptibility to most of the tested antibiotics with the exception of amikacin (30 μg) and erythromycin (15 μg). It is important to investigate the location of the antibiotic resistance genes and to determine their potential transfer prior to the commercial use of these isolates. This study facilitates an understanding of the differences in antibiotic resistance profiles among various LAB strains and establishes a basis for optimally selecting probiotics to manufacture high-quality fermented products.

**Table 1:** Antibiotic susceptibility profile of selected LAB isolates.

Strains	Ampicillin (10 μg)	Penicillin (10 μg)	Amoxycillin (10 μg)	Norfloxacin (10 μg)	Levofloxacin (5 μg)	Gentamicin (120 μg)	Streptomycin (10 μg)	Amikacin (30 μg)	Erythromycin (15 μg)
*W. cibaria* Ba-4	S	R	S	R	IS	S	R	R	R
*L. mesenteroides* B-25	R	R	S	R	S	S	R	R	R
*L. lactis* B-24	S	R	S	S	S	S	R	R	S
*P. pentosaceus Ca-4*	IS	R	S	R	IS	S	R	R	R
*E. hirae* G-3	IS	S	S	IS	IS	S	IS	R	R
*L. garvieae* Pa-2	S	R	S	IS	IS	IS	IS	R	R
*W. confusa* Ra-2	S	R	S	R	S	S	IS	R	R
*L. plantarum* CICC20265	S	R	S	R	R	S	R	R	R
*S. thermophilus* CICC6220	S	R	S	R	IS	S	R	R	R

*Strains showing resistant (R), susceptibility (S) and intermediate susceptibility (IS).*

### Surface Hydrophobicity

**FIGURE 6:**
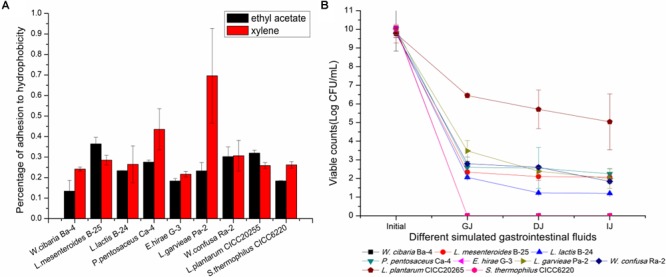
Evaluation of hydrophobicity to ethyl acetate and xylene and survival of simulated gastrointestinal digestion of the nine selected LAB strains. **(A)** Percentage of hydrophobicity. **(B)** Survival capacity of LAB strains during simulated gastrointestinal digestion. GJ, gastric juice; DJ, duodenal juice; IJ, intestinal juice.

Adhesion to the intestinal epithelial mucosa is related to many beneficial functions that are attributed to probiotics (Dunne et al., 2001). This is a complicated process that involves contact of bacteria with the intestinal mucosa surface and is influenced by multiple factors. In previous studies, the cell adhesion capability of *Streptococcus, Lactobacillus*, and *Bifidobacterium* species has been assessed by testing their adhesion to hydrocarbons. A positive correlation between adhesion ability and hydrophobicity has been observed (Wadstrom et al., 1987; Colloca et al., 2000; Nikolic et al., 2010). Some researchers have proposed that surface hydrophobicity could be used to identify *Bifidobacterium* species with adhesion potential to enterocytes (Del Re et al., 2000). Therefore, the reliability of the use of bacterial adhesion to hydrophobic compounds to measure the adhesion ability of LAB is clear (Vinderola et al., 2004). In our study, the phase separation of bacterial cells between the aqueous phase and ethyl acetate and xylene is shown in Figure 6A. The data demonstrated adhesion percentages of 13.4 ± 5.2% ∼ 36.4 ± 3.2% and 21.6 ± 1.4% ∼ 69.6 ± 2.3% to ethyl acetate and xylene, respectively, supporting the hypothesis that the cells possessed good adhesion properties. Previous reports have shown that the percentage of adhesion to ethyl acetate and xylene of *Propionibacterium* species ranged from 7.0 ± 2.8 to 71.0 ± 2.1% and from 2.0 ± 1.0 to 79.0 ± 1.6%, respectively (Darilmaz et al., 2012). Similar reports have shown that the binding percentage of *Bifidobacterium* and *Lactobacillus* to xylene was in the range of 17.4 ± 8.5% ∼ 75.2 ± 9.0% and 13.5 ± 5.0% ∼ 67.1 ± 10.7%, respectively (Collado et al., 2008). In this study, the most hydrophobic strains were *L. mesenteroides* B-25 (36.4 ± 3.2% to ethyl acetate) and *L. garvieae* Pa-2 (69.6 ± 2.3% to xylene). Moreover, *L. garvieae* Pa-2 presented binding proportions of 23.3 ± 4.0 and 69.6 ± 2.3% to ethyl acetate and xylene, respectively, which represented a significant difference. Our results revealed a great heterogeneity in adhesion to hydrophobic compounds. High or low affinity for a solvent did not exclude simultaneous affinity for the other solvent, suggesting that the cell surface was very complex. This may be due to the presence of proteins or polysaccharides on the cell surface leading to differences in hydrophobicity (Walker, 2008; Giri et al., 2018). The cell surface hydrophobicity test results can be used for preliminary screening in order to identify probiotic bacteria that are suitable for human or animal use.

### Response to Simulated Gastrointestinal Tract Conditions

Tolerance to low pH and bile salts during transit through the gastrointestinal tract is essential for LAB to survive, grow, and exert their beneficial functions (Jena et al., 2013). The loss of viability after exposure to simulated gastrointestinal tract conditions has been reported in several previous studies (Santos et al., 2016; Freire et al., 2017). The survival rate or loss of viability was calculated by a comparison of bacterial counts during the gastrointestinal transit *in vitro*. As shown in Figure 6B, *P. pentosaceus* Ca-4 and *L. mesenteroides* B-25 exhibited the highest survival rates after the gastrointestinal transit of 22.4 ± 2.6 and 21.2 ± 1.4%, respectively. However, the viable population only maintained 3.0 Log CFU/mL, which was significantly lower than that of *L. plantarum* CICC20265. *W. cibaria* Ba-4, *E. hirae* G-3, and *S. thermophilus* CICC6220 lost their viability during the transit. The LAB present in fermented FVJs must sustain their viability during gastrointestinal transit and achieve eventual engraftment in the host gut mucosa (Ranadheera et al., 2012). According to the literature, several strains exhibit different cell survival rates under harsh environmental conditions. For instance, cell counts of 6.40 Log CFU/g of *Lactobacillus bulgaricus*, 8.70 Log CFU/mL of *L. casei* DN-114 001, and 5.86 Log CFU/g of *P. pentosaceus* Q3 remained after gastrointestinal transit. Survival rates of 0.1–40% for *L. lactis* and 36.6% for *L. mesenteroides* IM082 were reported, which suggests that microencapsulation and other protective technologies may be beneficial for extending the application of probiotics (Oozeer et al., 2004; Mainville et al., 2005; Dobson et al., 2011; Jensen et al., 2012; Chen et al., 2017). After transiting through simulated GJ for 3 h, the *Bifidobacterium* species exhibited a viable bacterial cell count of 7.32 Log CFU/mL, with a survival rate of 72.1%, and these results may be associated with the anaerobic fermentation characteristics of the *Bifidobacterium* species (Watson et al., 2008). Notably, the colonization level and the capacity to remain in the gastrointestinal tract were somewhat inconsistent among different strains. Hence, it is important to highlight that the isolates identified from broccoli juice did not show specific superiority when compared with other strains, although some studies have reported that strain variation as well as an appropriate carrier food matrix can potentially improve the survival of probiotics in the presence of simulated gastric and small intestinal juices (Saxelin et al., 2010; Ranadheera et al., 2012). Fruits and vegetables are valuable nutrient sources, making them ideal substrates for growing probiotics (Shori, 2016). Researchers revealed that the composition of the carrier food matrix such as fat content may provide additional protection for probiotic species (Pigeon et al., 2002; Vinderola and Reinheimer, 2003). Even though the isolates in this study were all indigenous phytogenic strains, there were observable differences in tolerance to acidic conditions and bile salts in terms of different survival rates during passage through the gastrointestinal tract. The results showed that the application of probiotic cultures in different food matrices could represent a great challenge for the viability of probiotics. It is essential for the isolated strains to have a protection system to withstand the low pH in the stomach and digestive enzymes and bile of the small intestine (Jensen et al., 2012). The findings of this study suggest that adequate measurement of probiotic potential LAB starters should be carried out in the intended carrier foods. In summary, based on our study results, potential LAB starters used to obtain reliable and controlled fermentation processes can be selected from the isolates of autochthonous microbiota of raw FVJs, for example, *L. garvieae* Pa-2 (GenBank accession number: MH198321), *P. pentosaceus* Ca-4 (GenBank accession number: MH198320), and *L. mesenteroides* B-25 (GenBank accession number: MH198322).

## Conclusion

Both the traditional culture-dependent method and molecular technique were used to determine the composition of LAB populations in fermented FVJs. A wide diversity of autochthonous bacterial communities was identified among the five fermented FVJs, namely, broccoli, ginger, pitaya, cherry, and radish juices. The fermentation characteristics of strains in broccoli juice, as well as their antibiotic resistance, hydrophobic properties, and survivability in the simulated gastrointestinal tract environment, which are all important factors that influence the efficacy of probiotics, were also investigated. The results indicated similarities and differences in bacterial abundance between the various fermented products, with isolated indigenous microbes present on the fruit and vegetable surface, as well as inoculated commercial species, having potential use in the processing of fermented FVJs.

The use of indigenous microbes and appropriate fermentation conditions are crucial for the elaboration of high-quality fermented FVJs. The species obtained in this study demonstrated their potential to be used as starter cultures to overcome unstable and/or unmanageable fermentation conditions encountered in the production of FVJs. Further investigations will aim to better understand the mechanisms underlying the observed diversity among different materials. In addition, further studies are still required to clarify how the endogenous microbiome can affect the properties of fermented juices and to identify the bacteria responsible for the quality of fermentation foods. Such research will aid in the development of functional autochthonous starters and help to diversify the availability of processed high-quality fruit and vegetable products.

## Author Contributions

JW and XL conceived and designed the experiments. XX wrote the paper. XX, DL, and YB revised the manuscript and performed the experiments.

## Conflict of Interest Statement

The authors declare that the research was conducted in the absence of any commercial or financial relationships that could be construed as a potential conflict of interest.

## Footnotes

1 http://drive5.com/uparse/

2 http://rdp.cme.msu.edu/

3 http://blast.ncbi.nlm.nih.gov/Blast.cgi

## References

[B1] Adewumi G. A. Oguntoyinbo F. A. Keisam S. Romi W. Jeyaram K. (2013). Combination of culture-independent and culture-dependent molecular methods for the determination of bacterial community of iru, a fermented *Parkia biglobosa* seeds. *Front. Microbiol.* 3:436. 10.3389/Fmicb.2012.00436 23316189 PMC3539807

[B2] Altschul S. F. Gish W. Miller W. Myers E. W. Lipman D. J. (1990). Basic local alignment search tool. *J. Mol. Biol.* 215 403–410. 10.1016/S0022-2836(05)80360-2 2231712

[B3] Ammor M. S. Florez A. B. Mayo B. (2007). Antibiotic resistance in non-enterococcal lactic acid bacteria and *Bifidobacteria*. *Food Microbiol.* 24 559–570. 10.1016/j.fm.2006.11.001 17418306

[B4] Aneja K. R. Dhiman R. Aggarwal N. K. Kumar V. Kaur M. (2014). Microbes associated with freshly prepared juices of citrus and carrots. *Int. J. Food Sci.* 2014:408085. 10.1155/2014/408085 26904628 PMC4745523

[B5] Armah C. N. Derdemezis C. Traka M. H. Dainty J. R. Doleman J. F. Saha S. (2015). Diet rich in high glucoraphanin broccoli reduces plasma LDL cholesterol: evidence from randomised controlled trials. *Mol. Nutr. Food Res.* 59 918–926. 10.1002/mnfr.201400863 25851421 PMC4692095

[B6] Baruah R. Maina N. H. Katina K. Juvonen R. Goyal A. (2017). Functional food applications of dextran from *Weissella cibaria* RBA12 from pummelo (*Citrus maxima*). *Int. J. Food Microbiol.* 242 124–131. 10.1016/j.ijfoodmicro.2016.11.012 27992769

[B7] Bautista-Gallego J. Arroyo-Lopez F. N. Rantsiou K. Jimenez-Diaz R. Garrido-Fernandez A. Cocolin L. (2013). Screening of lactic acid bacteria isolated from fermented table olives with probiotic potential. *Food Res. Int.* 50 135–142. 10.1016/j.foodres.2012.10.004

[B8] Ben Omar N. Ampe F. (2000). Microbial community dynamics during production of the Mexican fermented maize dough pozol. *Appl. Environ. Microbiol.* 66 3664–3673. 10.1128/Aem.66.9.3664-3673.2000 10966374 PMC92204

[B9] Ben Omar N. Castro A. Lucas R. Abriouel H. Yousif N. M. K. Franz C. M. A. P. (2004). Functional and safety aspects of enterococci isolated from different Spanish foods. *Syst. Appl. Microbiol.* 27 118–130. 10.1078/0723-2020-00248 15053328

[B10] Berenbaum F. (2014). Does broccoli protect from osteoarthritis? *Joint Bone Spine* 81 284–286. 10.1016/j.jbspin.2014.04.001 24956981

[B11] Bokulich N. A. Lewis Z. T. Boundy-Mills K. Mills D. A. (2016). A new perspective on microbial landscapes within food production. *Curr. Opin. Biotechnol.* 37 182–189. 10.1016/j.copbio.2015.12.008 26773388 PMC4913695

[B12] Chaovanalikit A. Wrolstad R. E. (2004). Total anthocyanins and total phenolics of fresh and processed cherries and their antioxidant properties. *J. Food Sci.* 69 C67–C72. 10.1111/j.1365-2621.2004.tb17858.x

[B13] Charteris W. P. Kelly P. M. Morelli L. Collins J. K. (1998). Antibiotic susceptibility of potentially probiotic *Lactobacillus species*. *J. Food Prot.* 61 1636–1643. 10.4315/0362-028x-61.12.1636 9874341

[B14] Chen H. Y. Li X. Y. Liu B. J. Meng X. H. (2017). Microencapsulation of *Lactobacillus bulgaricus* and survival assays under simulated gastrointestinal conditions. *J. Funct. Foods* 29 248–255. 10.1016/j.jff.2016.12.015

[B15] Choi I. K. Jung S. H. Kim B. J. Park S. Y. Kim J. Han H. U. (2003). Novel Leuconostoc citreum starter culture system for the fermentation of kimchi, a fermented cabbage product. *Antonie Van Leeuwenhoek* 84 247–253. 10.1023/A:1026050410724 14574101

[B16] Collado M. C. Meriluoto J. Salminen S. (2008). Adhesion and aggregation properties of probiotic and pathogen strains. *Eur. Food Res. Technol.* 226 1065–1073. 10.1007/s00217-007-0632-x

[B17] Colloca M. E. Ahumada M. C. Lopez M. E. Nader-Macias M. E. (2000). Surface properties of lactobacilli isolated from healthy subjects. *Oral Dis.* 6 227–233. 10.1111/j.1601-0825.2000.tb00118.x 10918560

[B18] Daily J. W. Zhang X. Kim D. S. Park S. (2015). Efficacy of ginger for alleviating the symptoms of primary dysmenorrhea: a systematic review and meta-analysis of randomized clinical trials. *Pain Med.* 16 2243–2255. 10.1111/pme.12853 26177393

[B19] Darilmaz D. O. Beyatli Y. Yuksekdag Z. N. (2012). Aggregation and hydrophobicity properties of 6 dairy propionibacteria strains isolated from homemade Turkish cheeses. *J. Food Sci.* 77 M20–M24. 10.1111/j.1750-3841.2011.02438.x 22260114

[B20] Davis C. (2014). Enumeration of probiotic strains: review of culture-dependent and alternative techniques to quantify viable bacteria. *J. Microbiol. Methods* 103 9–17. 10.1016/j.mimet.2014.04.012 24814752

[B21] Del Re B. Sgorbati B. Miglioli M. Palenzona D. (2000). Adhesion, autoaggregation and hydrophobicity of 13 strains of *Bifidobacterium longum*. *Lett. Appl. Microbiol.* 31 438–442. 10.1046/j.1365-2672.2000.00845.x 11123552

[B22] Di Cagno R. Coda R. De Angelis M. Gobbetti M. (2013). Exploitation of vegetables and fruits through lactic acid fermentation. *Food Microbiol.* 33 1–10. 10.1016/j.fm.2012.09.003 23122495

[B23] Di Cagno R. Filannino P. Vincentini O. Lanera A. Cavoski I. Gobbetti M. (2016). Exploitation of *Leuconostoc mesenteroides* strains to improve shelf life, rheological, sensory and functional features of prickly pear (*Opuntia ficus-indica* L.) fruit puree. *Food Microbiol.* 59 176–189. 10.1016/j.fm.2016.06.009 27375258

[B24] Di Cagno R. Surico R. F. Paradiso A. De Angelis M. Salmon J. C. Buchin S. (2009a). Effect of autochthonous lactic acid bacteria starters on health-promoting and sensory properties of tomato juices. *Int. J. Food Microbiol.* 128 473–483. 10.1016/j.ijfoodmicro.2008.10.017 19028404

[B25] Di Cagno R. Surico R. F. Minervini G. De Angelis M. Rizzello C. G. Gobbetti M. (2009b). Use of autochthonous starters to ferment red and yellow peppers (*Capsicum annum* L.) to be stored at room temperature. *Int. J. Food Microbiol.* 130 108–116. 10.1016/j.ijfoodmicro.2009.01.019 19217182

[B26] Di Cagno R. Surico R. F. Siragusa S. De Angelis M. Paradiso A. Minervini F. (2008). Selection and use of autochthonous mixed starter for lactic acid fermentation of carrots, French beans or marrows. *Int. J. Food Microbiol.* 127 220–228. 10.1016/j.ijfoodmicro.2008.07.010 18710789

[B27] Ding W. R. Shi C. Chen M. Zhou J. W. Long R. J. Guo X. S. (2017). Screening for lactic acid bacteria in traditional fermented Tibetan yak milk and evaluating their probiotic and cholesterol-lowering potentials in rats fed a high-cholesterol diet. *J. Funct. Foods* 32 324–332. 10.1016/j.jff.2017.03.021

[B28] Dobson A. Crispie F. Rea M. C. O’Sullivan O. Casey P. G. Lawlor P. G. (2011). Fate and efficacy of lacticin 3147-producing *Lactococcus lactis* in the mammalian gastrointestinal tract. *FEMS Microbiol. Ecol.* 76 602–614. 10.1111/j.1574-6941.2011.01069.x 21314706

[B29] Dunne C. O’Mahony L. Murphy L. Thornton G. Morrissey D. O’Halloran S. (2001). In vitro selection criteria for probiotic bacteria of human origin: correlation with in vivo findings. *Am. J. Clin. Nutr.* 73 386s–392s. 10.1093/ajcn/73.2.386s 11157346

[B30] Ellis R. J. Morgan P. Weightman A. J. Fry J. C. (2003). Cultivation-dependent and -independent approaches for determining bacterial diversity in fleavy-metal-contaminated soil. *Appl. Environ. Microbiol.* 69 3223–3230. 10.1128/Aem.69.6.3223-3230.2003 12788719 PMC161537

[B31] Ercolini D. Pontonio E. De Filippis F. Minervini F. La Storia A. Gobbetti M. (2013). Microbial ecology dynamics during rye and wheat sourdough preparation. *Appl. Environ. Microbiol.* 79 7827–7836. 10.1128/Aem.02955-13 24096427 PMC3837820

[B32] Espirito-Santo A. P. Carlin F. Renard C. M. G. C. (2015). Apple, grape or orange juice: which one offers the best substrate for Lactobacilli growth? – A screening study on bacteria viability, superoxide dismutase activity, folates production and hedonic characteristics. *Food Res. Int.* 78 352–360. 10.1016/j.foodres.2015.09.014 28433303

[B33] European Food Safety Authority [EFSA]. (2012). Guidance on the assessment of bacterial susceptibility to antimicrobials of human and veterinary importance. *EFSA J.* 10:2740.

[B34] Fan S. Breidt F. Price R. Perez-Diaz I. (2017). Survival and growth of probiotic lactic acid bacteria in refrigerated pickle products. *J. Food Sci.* 82 167–173. 10.1111/1750-3841.13579 27984668

[B35] Freire A. L. Ramos C. L. Souza P. N. D. Cardoso M. G. B. Schwan R. F. (2017). Nondairy beverage produced by controlled fermentation with potential probiotic starter cultures of lactic acid bacteria and yeast. *Int. J. Food Microbiol.* 248 39–46. 10.1016/j.ijfoodmicro.2017.02.011 28242421

[B36] Fusco V. Quero G. M. (2014). Culture-dependent and culture-independent nucleic-acid-based methods used in the microbial safety assessment of milk and dairy products. *Comprehen. Rev. Food Sci. Food Saf.* 13 493–537. 10.1111/1541-4337.12074 33412709

[B37] Fusco V. Quero G. M. Cho G. S. Kabisch J. Meske D. Neve H. (2015). The genus *Weissella*: taxonomy, ecology and biotechnological potential. *Front. Microbiol.* 6:155 10.3389/Fmicb.2015.00155 PMC4362408 25852652

[B38] Galvez A. Abriouel H. Lopez R. L. Ben Omar N. (2007). Bacteriocin-based strategies for food biopreservation. *Int. J. Food Microbiol.* 120 51–70. 10.1016/j.ijfoodmicro.2007.06.001 17614151

[B39] Garcia-Cruz L. Duenas M. Santos-Buelgas C. Valle-Guadarrama S. Salinas-Moreno Y. (2017). Betalains and phenolic compounds profiling and antioxidant capacity of pitaya (*Stenocereus* spp.) fruit from two species (*S. Pruinosus* and *S. stellatus*). *Food Chem.* 234 111–118. 10.1016/j.foodchem.2017.04.174 28551213

[B40] Gibbons J. G. Rinker D. C. (2015). The genomics of microbial domestication in the fermented food environment. *Curr. Opin. Genet. Dev.* 35 1–8. 10.1016/j.gde.2015.07.003 26338497 PMC4695309

[B41] Giello M. La Storia A. De Filippis F. Ercolini D. Villani F. (2018). Impact of *Lactobacillus curvatus* 54M16 on microbiota composition and growth of *Listeria monocytogenes* in fermented sausages. *Food Microbiol.* 72 1–15. 10.1016/j.fm.2017.11.003 29407386

[B42] Giri S. S. Sen S. S. Saha S. Sukumaran V. Park S. C. (2018). Use of a potential probiotic, *Lactobacillus plantarum* L7, for the preparation of a rice-based fermented beverage. *Front. Microbiol.* 9:473. 10.3389/Fmicb.2018.00473 29593702 PMC5861207

[B43] Granato D. Branco G. F. Nazzaro F. Cruz A. G. Faria J. A. F. (2010). Functional foods and nondairy probiotic food development: trends, concepts, and products. *Comprehen. Rev. Food Sci. Food Saf.* 9 292–302. 10.1111/j.1541-4337.2010.00110.x 33467814

[B44] Guo X. H. Kim J. M. Nam H. M. Park S. Y. Kim J. M. (2010). Screening lactic acid bacteria from swine origins for multistrain probiotics based on in vitro functional properties. *Anaerobe* 16 321–326. 10.1016/j.anaerobe.2010.03.006 20304081

[B45] Hashimoto T. Ueda Y. Oi N. Sakakibara H. Piao C. Ashida H. (2006). Effects of combined administration of quercetin, rutin, and extract of white radish sprout rich in kaempferol glycosides on the metabolism in rats. *Biosci. Biotechnol. Biochem.* 70 279–281. 10.1271/Bbb.70.279 16428850

[B46] Herrero M. Mayo B. Gonzalez B. Suarez J. E. (1996). Evaluation of technologically important traits in lactic acid bacteria isolated from spontaneous fermentations. *J. Appl. Bacteriol.* 81 565–570. 10.1111/j.1365-2672.1996.tb03548.x

[B47] Jacob R. A. Spinozzi G. M. Simon V. A. Kelley D. S. Prior R. L. Hess-Pierce B. (2003). Consumption of cherries lowers plasma urate in healthy women. *J. Nutr.* 133 1826–1829. 10.1093/jn/133.6.1826 12771324

[B48] Jena P. K. Trivedi D. Thakore K. Chaudhary H. Giri S. S. Seshadri S. (2013). Isolation and characterization of probiotic properties of *Lactobacilli* isolated from rat fecal microbiota. *Microbiol. Immunol.* 57 407–416. 10.1111/1348-0421.12054 23773019

[B49] Jensen H. Grimmer S. Naterstad K. Axelsson L. (2012). In vitro testing of commercial and potential probiotic lactic acid bacteria. *Int. J. Food Microbiol.* 153 216–222. 10.1016/j.ijfoodmicro.2011.11.020 22177712

[B50] Jiang M. L. Zhang F. Wan C. X. Xiong Y. H. Shah N. P. Wei H. (2016). Evaluation of probiotic properties of *Lactobacillus plantarum* WLPL04 isolated from human breast milk. *J. Dairy Sci.* 99 1736–1746. 10.3168/jds.2015-10434 26805974

[B51] Jung J. Y. Lee S. H. Lee H. J. Jeon C. O. (2013). Microbial succession and metabolite changes during fermentation of saeu-jeot: traditional Korean salted seafood. *Food Microbiol.* 34 360–368. 10.1016/j.fm.2013.01.009 23541203

[B52] Karovicova J. Kohajdova Z. (2005). Lactic acid-fermented vegetable juices – Palatable and wholesome foods. *Chem. Papers* 59 143–148.

[B53] Kaymak H. C. Ozturk S. Ercisli S. Guvenc I. (2015). In vitro antibacterial activities of black and white radishes (*Raphanus sativus* L.). *Comptes Rend. L Acad. Bulgare Des. Sci.* 68 201–208.

[B54] Keck A. S. Qiao Q. Y. Jeffery E. H. (2003). Food matrix effects on bioactivity of broccoli-derived sulforaphane in liver and colon of F344 rats. *J. Agric. Food Chem.* 51 3320–3327. 10.1021/jf026189a 12744661

[B55] Kesmen Z. Yetiman A. E. Gulluce A. Kacmaz N. Sagdic O. Cetin B. (2012). Combination of culture-dependent and culture-independent molecular methods for the determination of lactic microbiota in sucuk. *Int. J. Food Microbiol.* 153 428–435. 10.1016/j.ijfoodmicro.2011.12.008 22209604

[B56] Kim D. O. Heo H. J. Kim Y. J. Yang H. S. Lee C. Y. (2005). Sweet and sour cherry phenolics and their protective effects on neuronal cells. *J. Agric. Food Chem.* 53 9921–9927. 10.1021/jf0518599 16366675

[B57] Klare I. Konstabel C. Werner G. Huys G. Vankerckhoven V. Kahlmeter G. (2007). Antimicrobial susceptibilities of *Lactobacillus, Pediococcus* and *Lactococcus* human isolates and cultures intended for probiotic or nutritional use. *J. Antimicrob. Chemotherpy* 59 900–912. 10.1093/jac/dkm035 17369278

[B58] Kohli D. K. Bachhawat A. K. (2003). CLOURE: clustal output reformatter, a program for reformatting ClustalX/ClustalW outputs for SNP analysis and molecular systematics. *Nucleic Acids Res.* 31 3501–3502. 10.1093/nar/gkg502 12824353 PMC168909

[B59] Kruth P. Brosi E. Fux R. Morike K. Gleiter C. H. (2004). Ginger-associated overanticoagulation by phenprocoumon. *Ann. Pharmacother.* 38 257–260. 10.1345/aph.1D225 14742762

[B60] Le Bourgeois P. Passerini D. Coddeville M. Guellerin M. Daveran-Mingot M. L. Ritzenthaler P. (2015). PFGE protocols to distinguish subspecies of *Lactococcus lactis*. *Methods Mol. Biol.* 1301 213–224. 10.1007/978-1-4939-2599-5_17 25862059

[B61] Lee H. W. Yoon S. R. Kim S. J. Lee H. M. Lee J. Y. Lee J. H. (2017). Identification of microbial communities, with a focus on foodborne pathogens, during kimchi manufacturing process using culture-independent and -dependent analyses. *Lwt-Food Sci. Technol.* 81 153–159. 10.1016/j.lwt.2017.04.001

[B62] Lee J. Jang J. C. Kim B. Kim J. Jeong G. J. Han H. G. (2004). Identification of *Lactobacillus sakei* and *Lactobacillus curvatus* by multiplex PCR-based restriction enzyme analysis. *J. Microbiol. Methods* 59 1–6. 10.1016/j.mimet.2004.05.004 15325747

[B63] Lee K. W. Park J. Y. Sa H. D. Jeong J. H. Jin D. E. Heo H. J. (2014). Probiotic properties of *Pediococcus* strains isolated from jeotgals, salted and fermented Korean sea-food. *Anaerobe* 28 199–206. 10.1016/j.anaerobe.2014.06.013 24979684

[B64] Lee S. W. Yang K. M. Kim J. K. Nam B. H. Lee C. M. Jeong M. H. (2012). Effects of white radish (*Raphanus sativus*) enzyme extract on hepatotoxicity. *Toxicol. Res.* 28 165–172. 10.5487/TR.2012.28.3.165 24278606 PMC3834419

[B65] Liao X. Y. Guo L. Q. Ye Z. W. Qiu L. Y. Gu F. W. Lin J. F. (2016). Use of autochthonous lactic acid bacteria starters to ferment mango juice for promoting its probiotic roles. *Prep. Biochem. Biotechnol.* 46 399–405. 10.1080/10826068.2015.1045615 26176886

[B66] Liu D. Q. Tong C. (2017). Bacterial community diversity of traditional fermented vegetables in China. *Lwt-Food Sci. Technol.* 86 40–48. 10.1016/j.lwt.2017.07.040

[B67] Mainville I. Arcand Y. Farnworth E. R. (2005). A dynamic model that simulates the human upper gastrointestinal tract for the study of probiotics. *Int. J. Food Microbiol.* 99 287–296. 10.1016/j.ijfoodmicro.2004.08.020 15808363

[B68] Medina E. Brenes M. Garcia-Garcia P. Romero C. de Castro A. (2018). Microbial ecology along the processing of Spanish olives darkened by oxidation. *Food Control* 86 35–41. 10.1016/j.foodcont.2017.10.035

[B69] Medina E. Ruiz-Bellido M. A. Romero-Gil V. Rodriguez-Gomez F. Montes-Borrego M. Landa B. B. (2016). Assessment of the bacterial community in directly brined Alorena de Malaga table olive fermentations by metagenetic analysis. *Int. J. Food Microbiol.* 236 47–55. 10.1016/j.ijfoodmicro.2016.07.014 27442850

[B70] M’hir S. Minervini F. Di Cagno R. Chammem N. Hamdi M. (2012). Technological, functional and safety aspects of enterococci in fermented vegetable products: a mini-review. *Ann. Microbiol.* 62 469–481. 10.1007/s13213-011-0363-x

[B71] Moreno D. A. Carvajal M. Lopez-Berenguer C. Garcia-Viguera C. (2006). Chemical and biological characterisation of nutraceutical compounds of broccoli. *J. Pharm. Biomed. Anal.* 41 1508–1522. 10.1016/j.jpba.2006.04.003 16713696

[B72] Motato K. E. Milani C. Ventura M. Valencia F. E. Ruas-Madiedo P. Delgado S. (2017). Bacterial diversity of the Colombian fermented milk “Suero Costeno” assessed by culturing and high-throughput sequencing and DGGE analysis of 16S rRNA gene amplicons. *Food Microbiol.* 68 129–136. 10.1016/j.fm.2017.07.011 28800820

[B73] Mousavi Z. E. Mousavi S. M. Razavi S. H. Emam-Djomeh Z. Kiani H. (2011). Fermentation of pomegranate juice by probiotic lactic acid bacteria. *World J. Microbiol. Biotechnol.* 27 123–128. 10.1007/s11274-010-0436-1 28253999

[B74] Nam Y. D. Lee S. Y. Lim S. I. (2012). Microbial community analysis of Korean soybean pastes by next-generation sequencing. *Int. J. Food Microbiol.* 155 36–42. 10.1016/j.ijfoodmicro.2012.01.013 22305887

[B75] Nematollahi A. Sohrabvandi S. Mortazavian A. M. Jazaeri S. (2016). Viability of probiotic bacteria and some chemical and sensory characteristics in cornelian cherry juice during cold storage. *Electron. J. Biotechnol.* 21 49–53. 10.1016/j.ejbt.2016.03.001

[B76] Nicomrat D. Chamutpong S. (2016). Application of microbial community for enhancing nutritional and appealing fermented juice. *Appl. Mech. Mater.* 848 131–134. 10.4028/www.scientific.net/AMM.848.131

[B77] Nikolic M. Jovcic B. Kojic M. Topisirovic L. (2010). Surface properties of *Lactobacillus* and *Leuconostoc* isolates from homemade cheeses showing auto-aggregation ability. *Eur. Food Res. Technol.* 231 925–931. 10.1007/s00217-010-1344-1

[B78] Ong Y. Y. Tan W. S. Rosfarizan M. Chan E. S. Tey B. T. (2012). Isolation and identification of lactic acid bacteria from fermented red dragon fruit juices. *J. Food Sci.* 77 M560–M564. 10.1111/j.1750-3841.2012.02894.x 22924854

[B79] Oozeer R. Mater D. D. G. Goupil-Feuillerat N. Corthier G. (2004). Initiation of protein synthesis by a labeled derivative of the *Lactobacillus casei* DN-114 001 strain during transit from the stomach to the cecum in mice harboring human microbiota. *Appl. Environ. Microbiol.* 70 6992–6997. 10.1128/Aem.70.12.6992-6997.2004 15574892 PMC535174

[B80] Ouwehand A. C. Forssten S. Hibberd A. A. Lyra A. Stahl B. (2016). Probiotic approach to prevent antibiotic resistance. *Ann. Med.* 48 246–255. 10.3109/07853890.2016.1161232 27092975

[B81] Palatty P. L. Haniadka R. Valder B. Arora R. Baliga M. S. (2013). Ginger in the prevention of nausea and vomiting: a review. *Crit. Rev. Food Sci. Nutr.* 53 659–669. 10.1080/10408398.2011.553751 23638927

[B82] Park E. J. Chun J. Cha C. J. Park W. S. Jeon C. O. Bae J. W. (2012). Bacterial community analysis during fermentation of ten representative kinds of kimchi with barcoded pyrosequencing. *Food Microbiol.* 30 197–204. 10.1016/j.fm.2011.10.011 22265301

[B83] Park S. Ji Y. Park H. Lee K. Park H. Beck B. R. (2016). Evaluation of functional properties of lactobacilli isolated from Korean white kimchi. *Food Control* 69 5–12. 10.1016/j.foodcont.2016.04.037

[B84] Peng Q. N. Jiang S. M. Chen J. L. Ma C. C. Huo D. X. Shao Y. Y. (2018). Unique microbial diversity and metabolic pathwayfeatures of fermented vegetables from Hainan, China. *Front. Microbiol.* 9:399. 10.3389/Fmicb.2018.00399 29559966 PMC5845746

[B85] Perez-Cataluna A. Elizaquivel P. Carrasco P. Espinosa J. Reyes D. Wacher C. (2018). Diversity and dynamics of lactic acid bacteria in *Atole agrio*, a traditional maize-based fermented beverage from South-Eastern Mexico, analysed by high throughput sequencing and culturing. *Antonie Van Leeuwenhoek* 111 385–399. 10.1007/s10482-017-0960-1 29058140

[B86] Pigeon R. M. Cuesta E. P. Gilliland S. E. (2002). Binding of free bile acids by cells of yogurt starter culture bacteria. *J. Dairy Sci.* 85 2705–2710. 10.3168/jds.S0022-0302(02)74357-9 12487437

[B87] Pinto C. Pinho D. Cardoso R. Custodio V. Fernandes J. Sousa S. (2015). Wine fermentation microbiome: a landscape from different Portuguese wine appellations. *Front. Microbiol.* 6:905. 10.3389/Fmicb.2015.00905 26388852 PMC4555975

[B88] Ranadheera C. S. Evans C. A. Adams M. C. Baines S. K. (2012). In vitro analysis of gastrointestinal tolerance and intestinal cell adhesion of probiotics in goat’s milk ice cream and yogurt. *Food Res. Int.* 49 619–625. 10.1016/j.foodres.2012.09.007

[B89] Reina L. D. Perez-Diaz I. M. Breidt F. Azcarate-Peril M. A. Medina E. Butz N. (2015). Characterization of the microbial diversity in yacon spontaneous fermentation at 20 degrees C. *Int. J. Food Microbiol.* 203 35–40. 10.1016/j.ijfoodmicro.2015.03.007 25777679 PMC4587664

[B90] Rivera-Espinoza Y. Gallardo-Navarro Y. (2010). Non-dairy probiotic products. *Food Microbiol.* 27 1–11. 10.1016/j.fm.2008.06.008 19913684

[B91] Saitou N. Nei M. (1987). The neighbor-joining method – A new method for reconstructing phylogenetic trees. *Mol. Biol. Evol.* 4 406–425. 3447015 10.1093/oxfordjournals.molbev.a040454

[B92] Sanchez A. Garcia P. Rejano L. Brenes M. Garrido A. (1995). The effects of acidification and temperature during washing of Spanish-style green olives on the fermentation process. *J. Sci. Food Agric.* 68 197–202. 10.1002/jsfa.2740680210

[B93] Sanni A. I. (1993). The need for process optimization of African fermented foods and beverages. *Int. J. Food Microbiol.* 18 85–95. 10.1016/0168-1605(93)90213-Z 8494685

[B94] Santo A. P. D. Perego P. Converti A. Oliveira M. N. (2011). Influence of food matrices on probiotic viability – A review focusing on the fruity bases. *Trends Food Sci. Technol.* 22 377–385. 10.1016/j.tifs.2011.04.008

[B95] Santos T. T. Ornellas R. M. S. Arcucio L. B. Oliveira M. M. Nicoli J. R. Dias C. V. (2016). Characterization of lactobacilli strains derived from cocoa fermentation in the south of Bahia for the development of probiotic cultures. *Lwt-Food Sci. Technol.* 73 259–266. 10.1016/j.lwt.2016.06.003

[B96] Saxelin M. Lassig A. Karjalainen H. Tynkkynen S. Surakka A. Vapaatalo H. (2010). Persistence of probiotic strains in the gastrointestinal tract when administered as capsules, yoghurt, or cheese. *Int. J. Food Microbiol.* 144 293–300. 10.1016/j.ijfoodmicro.2010.10.009 21074284

[B97] Sharma C. Gulati S. Thakur N. Singh B. P. Gupta S. Kaur S. (2017). Antibiotic sensitivity pattern of indigenous lactobacilli isolated from curd and human milk samples. *Biotechnology* 7:53. 28444600 10.1007/s13205-017-0682-0 PMC5428095

[B98] Sheehan V. M. Ross P. Fitzgerald G. F. (2007). Assessing the acid tolerance and the technological robustness of probiotic cultures for fortification in fruit juices. *Innov. Food Sci. Emerg. Technol.* 8 279–284. 10.1016/j.ifset.2007.01.007

[B99] Shori A. B. (2016). Influence of food matrix on the viability of probiotic bacteria: a review based on dairy and non-dairy beverages. *Food Biosci.* 13 1–8. 10.1016/j.fbio.2015.11.001

[B100] Steinkraus K. H. (1997). Classification of fermented foods: worldwide review of household fermentation techniques. *Food Control* 8 311–317. 10.1016/S0956-7135(97)00050-9

[B101] Stoops J. Crauwels S. Waud M. Claes J. Lievens B. Van Campenhout L. (2016). Microbial community assessment of mealworm larvae (*Tenebrio molitor*) and grasshoppers (*Locusta migratoria* migratorioides) sold for human consumption. *Food Microbiol.* 53 122–127. 10.1016/j.fm.2015.09.010 26678139

[B102] Tamang J. P. Watanabe K. Holzapfel W. H. (2016). Review: diversity of microorganisms in global fermented foods and beverages. *Front. Microbiol.* 7:377. 10.3389/fmicb.2016.00377 27047484 PMC4805592

[B103] Tamura K. Peterson D. Peterson N. Stecher G. Nei M. Kumar S. (2011). MEGA5: molecular evolutionary genetics analysis using maximum likelihood, evolutionary distance, and maximum parsimony methods. *Mol. Biol. Evol.* 28 2731–2739. 10.1093/molbev/msr121 21546353 PMC3203626

[B104] Trząskowska M. Gasentzer P. (2016). Effects of probiotic *Lactobacillus rhamnosus* LOCK900 on the *Staphylococcus aureus* survival in carrot juice. *J. Food Saf.* 36 571–576. 10.1111/jfs.12278

[B105] Valerio F. De Bellis P. Lonigro S. L. Morelli L. Visconti A. Lavermicocca P. (2006). In vitro and in vivo survival and transit tolerance of potentially probiotic strains carried by artichokes in the gastrointestinal tract. *Appl. Environ. Microbiol.* 72 3042–3045. 10.1128/Aem.72.4.3042-3045.2006 16598015 PMC1449069

[B106] Vanhaecke E. Pijck J. (1988). Bioluminescence assay for measuring the number of bacteria adhering to the hydrocarbon phase in the bath test. *Appl. Environ. Microbiol.* 54 1436–1439. 16347654 10.1128/aem.54.6.1436-1439.1988 PMC202675

[B107] Vinderola C. G. Medici M. Perdigon G. (2004). Relationship between interaction sites in the gut, hydrophobicity, mucosal immunomodulating capacities and cell wall protein profiles in indigenous and exogenous bacteria. *J. Appl. Microbiol.* 96 230–243. 10.1046/j.1365-2672.2004.02158.x 14723684

[B108] Vinderola C. G. Reinheimer J. A. (2003). Lactic acid starter and probiotic bacteria: a comparative “in vitro” study of probiotic characteristics and biological barrier resistance. *Food Res. Int.* 36 895–904. 10.1016/S0963-9969(03)00098-X

[B109] Wadstrom T. Andersson K. Sydow M. Axelsson L. Lindgren S. Gullmar B. (1987). Surface-properties of *Lactobacilli* isolated from the small-intestine of pigs. *J. Appl. Bacteriol.* 62 513–520. 10.1111/j.1365-2672.1987.tb02683.x 3305459

[B110] Walker W. A. (2008). Mechanisms of action of probiotics. *Clin. Infect. Dis.* 46 S87–S91. 10.1086/523335 18181730

[B111] Wang Y. Y. Cao P. H. Wang L. Zhao Z. Y. Chen Y. L. Yang Y. X. (2017). Bacterial community diversity associated with different levels of dietary nutrition in the rumen of sheep. *Appl. Microbiol. Biotechnol.* 101 3717–3728. 10.1007/s00253-017-8144-5 28175950

[B112] Wang Z. X. Shao Y. Y. (2018). Effects of microbial diversity on nitrite concentration in pao cai, a naturally fermented cabbage product from China. *Food Microbiol.* 72 185–192. 10.1016/j.fm.2017.12.003 29407396

[B113] Watson D. Sleator R. D. Hill C. Gahan C. G. M. (2008). Enhancing bile tolerance improves survival and persistence of *Bifidobacterium* and *Lactococcus* in the murine gastrointestinal tract. *BMC Microbiol.* 8:176. 10.1186/1471-2180-8-176 18844989 PMC2577680

[B114] Wichienchot S. Jatupornpipat M. Rastall R. A. (2010). Oligosaccharides of pitaya (dragon fruit) flesh and their prebiotic properties. *Food Chem.* 120 850–857. 10.1016/j.foodchem.2009.11.026

[B115] Yoon S. R. Kim S. H. Lee H. W. Ha J. H. (2017). A novel method to rapidly distinguish the geographical origin of traditional fermented-salted vegetables by mass fingerprinting. *PLoS One* 12:e0188217. 10.1371/journal.pone.0188217 29149220 PMC5693415

